# Adherence to national food-based dietary guidelines and incidence of stroke: A cohort study of Danish men and women

**DOI:** 10.1371/journal.pone.0206242

**Published:** 2018-10-24

**Authors:** Sine Hammer Hansen, Kim Overvad, Camilla Plambeck Hansen, Christina Catherine Dahm

**Affiliations:** 1 Department of Public Health, Aarhus University, Aarhus, Denmark; 2 Department of Cardiology, Aalborg University Hospital, Aalborg, Denmark; 3 The Danish Clinical Registries, Aarhus N, Denmark; University of Zurich, SWITZERLAND

## Abstract

**Background and purpose:**

National dietary guidelines are intended to promote primary prevention of lifestyle-related diseases, but little is known about their effectiveness in prevention of stroke.

**Methods:**

We used the Danish cohort Diet, Cancer and Health (n = 57 053) to investigate whether adherence to the Danish food-based dietary guidelines was associated with risk of stroke. Adherence was assessed by the Danish Dietary Guidelines Index, score 0 [no adherence] to 6 [complete adherence]. Cox proportional hazards models were used to estimate adjusted hazard ratios and 95% confidence intervals for stroke and subtypes of stroke in men and women separately.

**Results:**

Incident stroke was determined in 1357 men and 900 women during follow-up (median 12.5 years and 13.0 years, respectively). A higher Danish Dietary Guidelines Index score was inversely associated with total stroke in men but not in women. In men, a high Index score (≥4) was also inversely associated with total ischemic stroke (hazard ratio 0.75, 95% confidence interval 0.65–0.86), large-artery atherosclerosis (hazard ratio 0.63, 95% confidence interval 0.44–0.92) and small artery occlusion (hazard ratio 0.68, 95% confidence interval 0.54–0.84) compared to a low Index score (<4). In women, inverse associations were found for total ischemic stroke (hazard ratio 0.84, 95% confidence interval 0.72–0.98) and intracerebral hemorrhage (hazard ratio 0.64, 95% confidence interval 0.43–0.96).

**Conclusions:**

Our findings suggest that adherence to the Danish Dietary Guidelines is associated with a lower rate of stroke, and thus may be useful in primary prevention of disease.

## Introduction

According to World Health Organisation (WHO) estimates, stroke is the second leading cause of death in the developed world and the third leading cause of long-term disability. [1,2] Stroke survivors may experience mild to severe disability from the brain damage, and medical treatment, rehabilitation and the need for lifelong help make stroke a leading burden of disease. [2] Primary prevention is key to reducing this burden in the population. Modifiable risk factors, including diet, are impactful, explaining 90% of all strokes. [3,4]

The Danish national food-based dietary guidelines have been developed to promote primary prevention of nutrition-related diseases. [5] The most recent update in 2013 was based on the current scientific evidence for foods and risk of non-communicable diseases, and on the Nordic Nutrition Recommendations 2012, [5,6] and adherence to these guidelines is associated with a lower rate of myocardial infarction. [7] Few studies have investigated associations between national dietary guidelines and incidence of stroke. [8] A study investigating adherence to the Dutch national dietary guidelines observed no association with total stroke. [9] However, total stroke includes ischemic and hemorrhagic subtypes, which have different underlying etiologies. [3,4] The aims of this study were thus to investigate whether adherence to the Danish dietary guidelines was associated with lower rates of total stroke or subtypes of stroke. We hypothesized that associations would vary by sex, as age at first stroke and incidence of stroke subtypes differs for men and women.

## Methods

### Study design and population

The Danish cohort, Diet, Cancer and Health was established between December 1993 to May 1997, when 160 725 men and women aged 50–64 years living in the greater areas of Copenhagen and Aarhus, born in Denmark and with no previous cancer diagnoses registered in the Danish Cancer Registry were invited to participate. Exclusion criteria for the current study were cancer or stroke diagnoses before enrolment, missing information on diet, or missing information on covariates. Requests to access the dataset may be sent to the Danish Data Archive at https://www.sa.dk/en/services/dda-danish-data-archive/.

### Dietary data

At baseline the participants completed a mailed detailed 192-item semi-quantitative food frequency questionnaire (FFQ). The questionnaire was specifically developed for the study[10] and validated against weighed diet records. [11] Participants were asked about the average intake of different food and beverage items over the previous 12 months, within 12 possible categories ranging from never to eight or more times per day. [11] The FFQ was processed by optical scanning at the study centres and checked for reading errors and missing information. All unclear information was then clarified with the participant during a computer guided interview. The software program FoodCalc was used to calculate average daily intake of foods and nutrients based on the Danish food composition tables, using standard sex-specific portion sizes. [11,12]

### The Danish Dietary Guidelines Index

We used an index developed previously to assess adherence to the food-based components of the Danish dietary guidelines 2013 (Table 1). [7] The Index comprises six components: fish, fruits and vegetables, whole grains, lean meat and cold-meat, saturated fat and sugar. Since the Danish dietary guidelines 2013 do not specify amounts of recommended saturated fat and sugar intake, the Nordic nutrition recommendations were used to set limits for these nutrients. [6] The guidelines ‘Choose low-fat dairy products’, ‘Eat food with less salt’, ‘Drink water’ and ‘Eat a variety of foods, but not too much, and be physically active’ were not included in the index for the following reasons: most milkfat is saturated, and thus intake of lower fat products is reflected in the component covering saturated fat; we were unable to distinguish between individual’s intakes of high- or low-salt versions of recipes on the FFQ; the guideline to drink water instead of soft drinks would be reflected in a lower intake of added sugar; and it is only possible to score highly on the Danish Dietary Guidelines Index if eating a variety of foods.[7]

**Table 1:** The official Danish dietary guidelines, issued by the Danish Ministry of food in 2013, and scoring on the Danish Dietary Guidelines Index.

Guideline components	Included in the Index	Index components	Criteria for minimum score	Criteria for maximum score
1. Eat a variety of food, not too much and be physically active.	✕			
2. Eat more fish.	✓	Minimum 350 g per week including 200 g fatty fish.	0 g fish per week	≥350 g fish per week including ≥200 g fatty fish.
3. Eat fruits and many vegetables.	✓	Minimum 600 g fruits and vegetables per day including up to 100 ml juice and minimum 300 g vegetables (excluding mushrooms and potatoes).	0 g fruits and vegetables per day	≥600 g fruit and vegetables per day including ≥300 g vegetables per day.
4. Choose whole grains.	✓	Minimum 75 g whole grains per day.	0 g whole grains per day	≥75 g whole grains per day.
5. Choose lean meat and cold-meat.	✓	Maximum 500 g red and processed meat per week.	≥1000 g red and processed meat per week	≤500 g red or processed meat per week.
6. Choose low-fat dairy products.	✕			
7. Eat less saturated fat.	✓	Maximum 10 E% from saturated fatty acids.*	≥20 E% from saturated fatty acids.	<10 E% from saturated fatty acids.
8. Eat food with less salt.	✕			
9. Eat less sugar.	✓	Maximum 10 E% from added sugar.*	≥20 E% from added sugar.	<10 E% from added sugar.
10. Drink water.	✕			

*E%, energy percentage. Maximum intake of saturated fat and sugar is taken from the Nordic Nutrition Recommendations 2012.

Adherence to each individual guideline contributed with a score from zero to one. For guidelines with a recommended minimum intake, scores were calculated as the ratio between the actual intake and the recommended intake, e.g. if an individual ate 60 g of wholegrain per day, the score was calculated as 60 g/75 g = score 0.8. If a guideline included more than one piece of advice, such as the fruit and vegetables guideline, the points were calculated for both pieces of advice and the individual score was multiplied with 0.5, thus a maximum score could only be achieved by full adherence to both pieces of advice. For guidelines with an upper limit of recommended intake, the score was calculated as one minus the difference between actual intake and recommended intake divided by the recommended intake, e.g. if an individual had an intake of 12% of energy from saturated fat the score was calculated as 1-(12-10/10) = score 0.8. For guidelines with a minimum limit, no further points were given for exceeding the minimum recommended intake. For guidelines with a recommended maximum, if an individual’s intake was double the recommended intake, zero points were given. Scores from the six components were summed to a total score from zero [no adherence] to six [complete adherence].[7]

### Covariates

At baseline, the participants filled in a background questionnaire concerning lifestyle factors and medical health, including information on physical activity, smoking habits, alcohol intake, educational level and history of hypercholesterolemia, hypertension and diabetes, and medication use. Leisure-time physical activity during the past year was reported as hours per week engaged in walking, gardening, housework, home maintenance, biking and sports during summer and winter. Moderate-to-vigorous leisure-time physical activity was calculated as the average number of hours per week spent on biking and sports during the whole year. All unclear answers were clarified in an interview with a lab technician, who also performed the physical examination, including measurements of height, weight and waist circumference. Height was measured to the nearest half centimetre when standing without shoes. Weight was measured to the nearest 0.1 kg using digital scales, with participants wearing only light clothes or underwear. Waist circumference was measured to the nearest 0.5 centimetre using a rigid measuring tape at the narrowest point between the lower rib and the iliac crest while standing. In cases of indeterminate waist narrowing, waist circumference was measured half way between the lower rib and the iliac crest.[13]

### Stroke classification

The outcome was first time stroke during follow-up. The Civil Registration System provides linkage to The Danish National Patient Register and The Causes of Deaths Register from which information on probable cases of nonfatal and fatal stroke (International Classification of Diseases 10 codes I60 (subarachnoid haemorrhage), I61 (intracerebral haemorrhage), I63 (cerebral infarction) and I64 (unspecified stroke)) was collected. [14,15] All cases were subsequently verified by individual review of medical journals by a physician with neurological experience. [14] Stroke was defined as a rapid onset of focal or global neurological deficit of a vascular origin, lasting >24 hours, either leading to death or else confirmed by a computed tomography or magnetic resonance imaging scan. Ischemic stroke was further sub-classified according to the Trial of Org 10172 in Acute Stroke Treatment (TOAST) classification system. [16] Analyses were conducted for total stroke, total haemorrhagic stroke, intracerebral haemorrhage, total ischemic stroke, large-artery atherosclerosis and small-artery occlusion.

### Statistical methods

Cox proportional hazards models, using age as the underlying timescale, were used to estimate hazard ratios and 95% confidence intervals for associations between the Danish Dietary Guidelines Index and incidence of stroke and subtypes of stroke. Participants were considered at risk from inclusion into the study until time of stroke, death due to other causes, emigration or 30^th^ December 2009.

The Danish Dietary Guidelines Index scores were categorized as <3, 3-<4, 4-<5 and ≥5 as previously; categories were included as a continuous variable to determine a p-value for trend. For the analyses of subtypes of stroke the Danish Dietary Guidelines Index categories were collapsed into <4 and ≥4 because of limited number of cases.

Potential confounders were chosen a priori based on previous studies. [3,4,17] Interaction between the Danish Dietary Guidelines Index and sex was investigated on the multiplicative scale using a Wald test for the interaction term. Sex was a statistically significant effect modifier (*P* = 0.036), thus analyses were conducted separately for men and women. Model 1a adjusted for age and enrolment date (quintiles). Model 1b additionally included physical activity (<30 or ≥30 minutes/day), alcohol intake (grams/day), smoking habits (never smoker, former smoker and current smoker, current smoker <15 cigarettes/day, 15–25 cigarettes/day, >25 cigarettes/day) and education (no vocational education, vocational or academic education <3 years, vocational or academic education 3–4 years, academic education >4 years). Model 2 additionally included BMI (kg/m2), waist circumference (cm) and history of hypertension, hypercholesterolemia and diabetes (yes, no, don´t know). Continuous variables were entered as restricted cubic splines with three knots (10th, 50th and 90th percentiles), then tested for linearity using Wald tests. Alcohol intake was not linearly associated with total stroke and was thus included as restricted cubic splines. Supplemental analyses stratifying model 1b by educational level, BMI, smoking, history of hypertension and history of hypercholesterolemia were performed for total stroke and total ischemic stroke.

Log-rank tests on Schoenfeld’s residuals and log-log plots were used to test proportional hazards assumption in models of The Danish Dietary Guidelines Index and total stroke. No substantial violations were found. Date of enrolment was statistically significantly associated with incidence of total stroke, and was thus included to fulfil the assumptions of independent entry.

Stata Version 14.2 was used. Reported tests are two-tailed and p-values below 0.05 were considered statistically significant.

### Ethics

The study complies with the current laws of Denmark, in which the study was performed. All procedures performed in studies involving human participants were in accordance with the ethical standards of the institutional and/or national research committee and with the 1964 Helsinki declaration and its later amendments or comparable ethical standards. Written informed consent was obtained from all individual participants included in the study.

The study was approved by the local ethical committees of Copenhagen and Frederiksberg Municipalities Municipalities (in Danish: "Den Videnskabsetiske komite for Københavns og Frederiksberg Kommuner") approval no.: (KF) 01-345/93, and the Danish Data Protection Agency.

## Results

**Fig 1:**
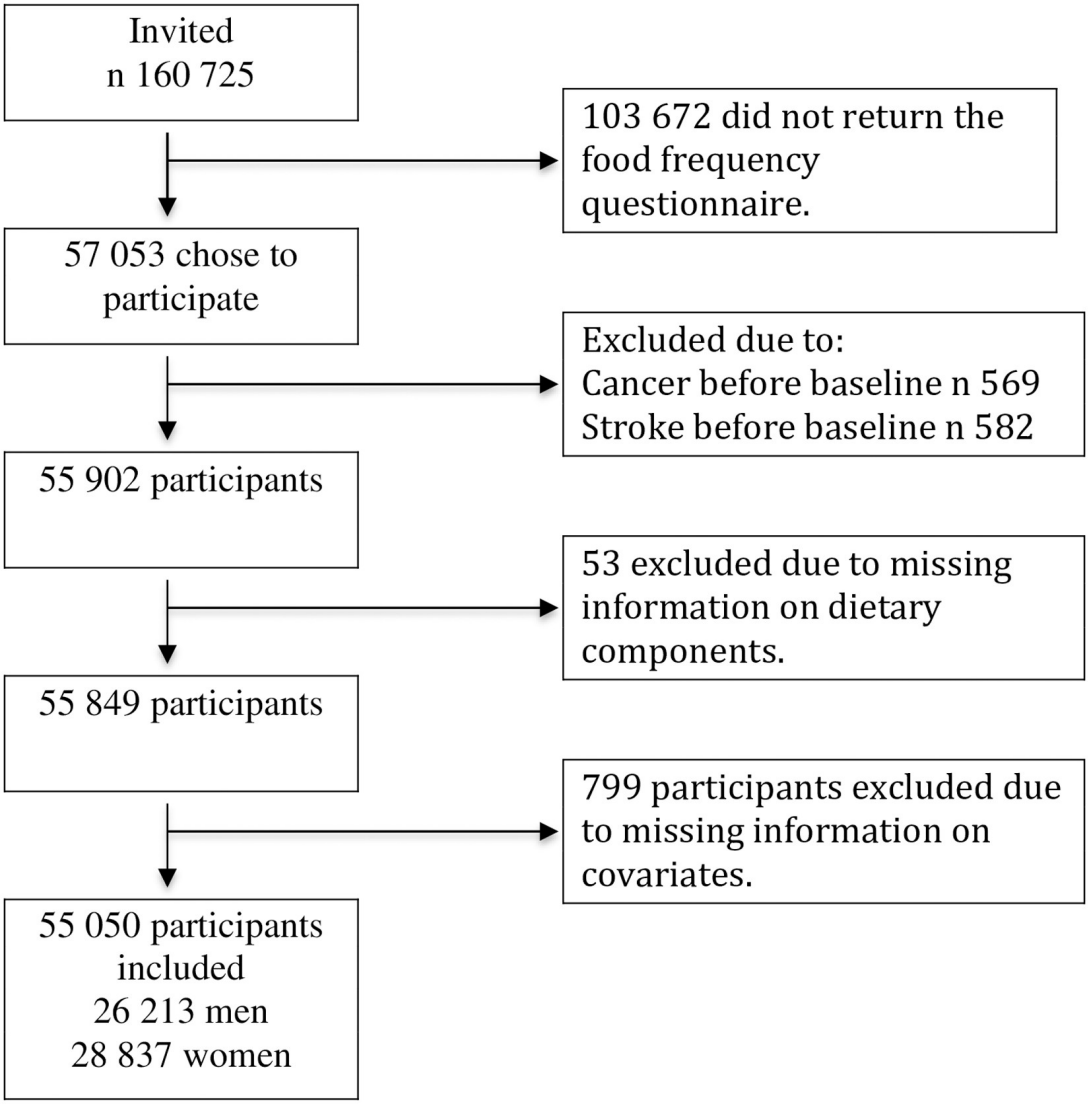
Flowchart of the study population from the Diet, Cancer and Health cohort.

A flow chart of the study population is shown in Fig 1. Of the 57 053 men and women who chose to participate, 569 were excluded due to a delay in registration of a cancer diagnosis before baseline in the Danish Cancer Registry; 582 participants were excluded due to a stroke diagnosis before baseline, 53 due to missing information on diet and 799 due to missing information on covariates. Thus, in this study a total of 55 050 participants were included (26 213 men and 28 837 women; median follow-up time 12.5 and 13.0 years, respectively). A first incident stroke was experienced by 1357 men and 900 women.

Baseline characteristics of the study population are shown in Table 2. Male cases were more likely to score fewer than 3 points on the Danish Dietary Guidelines Index and to have a lower intake of fruit and vegetables and a higher intake of red and processed meat than all men.

**Table 2:** Characteristics of the study population.

Characteristics	Men (n = 26 213)				Women (n = 28 837)			
	All men		Cases (n = 1357)		All women		Cases (n = 900)	
Dietary characteristics								
Danish Dietary Guidelines Index score, n(%)								
<3	5 693	21.7	386	28.5	2680	9.3	99	11.0
3-<4	12 721	48.5	659	48.6	11 276	39.1	408	45.3
4-<5	6 859	26.2	284	20.9	12 062	41.8	310	34.4
≥6	940	3.6	28	2.1	2819	9.8	83	9.2
Fish, g/week								
Total fish	292.3	85.1–691.8	189.6	81.4–675.0	246.8	74.6–591.0	259.9	77.5–641.4
Fatty fish	106.0	17.3–324.8	102.1	12.0–336.3	85.3	13.8–281.0	85.8	15.9–289.0
Vegetables, fruits and juice, g/day								
Vegetables	141.9	42.3–321.0	124.2	36.4–298.5	164.3	46.6–370.1	148.0	39.2–356.4
Fruits	117.8	18.2–420.1	104.4	14.6–387.2	172.3	30.9–508.6	157.9	27.0–491.6
Juice	8.6	0.1–103.7	8.4	0.1–103.4	9.07	0.1–120.4	8.7	0.1–129.0
Whole grains, g/day	42.4	11.9–86.6	39.5	9.9–83.7	34.2	10.6–75.8	32.5	9.1–73.0
Red and processed meat, g/week	971.9	466.3–1780.5	1009.4	487.7–1830.3	585.3	244.5–1096.7	611.3	268.5–1206.4
Saturated fat, E%*	13.8	9.5–17.9	14.1	9.5–18.2	13.1	8.6–17.6	13.5	8.6–17.8
Added sugar, E%*	5.5	1.7–13.7	5.4	1.5–13.6	6.1	2.0–14.8	6.0	1.7–14.4
Age, years	55.9	50.7–64.2	58.2	50.9–64.7	56.2	50.8–64.2	59.3	51.1–64.7
Physical activity, n(%)								
<30 min/day	16 264	62.1	908	66.9	17 026	59.0	577	64.1
≥30 min/day	9949	37.9	449	33.1	11 811	41.0	323	35.9
Smoking, n(%)								
Never	6776	25.9	276	20.3	12 662	43.9	263	29.9
Former	9067	34.6	404	29.8	6785	23.5	186	20.7
Current	10 370	39.6	677	49.9	939	32.6	451	50.1
<15 cigarettes/day	2780	10.6	147	10.8	4393	15.2	195	21.7
15–25 cigarettes/day	4581	17.5	329	24.2	4263	14.8	226	25.1
>25 cigarettes/day	3009	11.5	201	14.8	734	2.6	30	3.3
Alcohol intake, g/day	19.4	1.7–80.2	20.6	0.9–87.6	9.4	0.4–41.5	8.8	0.4–46.8
Vocational or academic education, n(%)								
None	2600	9.9	185	13.6	5534	19.2	214	23.8
<3 years	3559	13.6	191	14.1	9076	31.5	289	32.1
3–4 years	11 089	42.3	560	41.3	10972	38.1	306	34.0
>4 years	8965	34.2	421	31.0	3255	11.3	91	10.1
BMI, (kg/m^3^)	26.2	21.5–32.9	26.7	21.6–33.9	24.8	19.9–33.8	25.0	19.6–34.9
Waist circumference, cm	95	81–114	97	83–117	80	67–103	82	68–107
History of hypercholesterolemia, n(%)								
Yes	2241	8.6	160	11.8	1804	6.3	87	9.7
No	13 194	50.3	652	48.0	14 537	50.4	444	49.3
Don’t know	10 778	41.1	545	40.2	12 496	43.3	369	41.0
History of hypertension, n(%)								
Yes	3843	17.4	334	24.6	4938	17.1	292	32.4
No	18 174	69.3	798	58.8	20 918	72.5	523	58.1
Don’t know	4169	16.0	225	16.6	2981	10.4	85	9.5
History of diabetes, n(%)								
Yes	683	2.6	66	4.9	426	1.5	31	3.4
No	24 156	92.2	1203	88.6	27 283	94.6	827	91.9
Don’t know	1374	5.2	88	6.5	1128	3.9	42	4.7
Stroke subtypes, n(%) of cases								
Ischemic stroke			1147	84.5			713	79.2
Large-artery atherosclerosis			183	15.9			133	18.7
Cardioembolism			69	6.0			31	4.4
Stroke of other determined etiology			59	5.1			39	5.5
Stroke of undetermined etiology			331	28.9			180	25.3
Hemorrhagic stroke			200	14.8			184	20.5
Intracerebral hemorrhage			162	11.9			107	11.9
Subarachnoid hemorrhage			38	2.8			77	8.6
Unspecified stroke			10	0.7			3	0.3
Fatal stroke			87	6.4			71	7.9

*Energy percentage

Cases are participants who experienced a first time stroke during follow-up. Values are medians (5th and 95th percentiles) unless otherwise indicated.

Female cases were more likely to score 3-<4 points on the Danish Dietary Guidelines Index and to have a lower intake of fruit and vegetables, and a higher intake of red and processed meat and fish than all women.

Stroke cases, regardless of sex, were older at baseline, and less likely to be active ≥30 minutes a day than the total cohort. They were also more likely to report current smoking and a history of hypertension than the total cohort.

Covariate distributions for men and women according to the Index categories are shown in S1 and S2 Tables. Participants scoring ≥5 points were older and had lower BMI and waist circumference than those scoring <3, regardless of sex. They were more likely to be physically active ≥30 min/day, have higher education, be non-smokers and have a report a diagnosis or use of medicine related to hypercholesterolemia, hypertension and diabetes than men with a score <3, but were less likely to respond don’t know to the diseases.

### Associations with total stroke

Table 3 shows results for total stroke among men and women respectively. The hazard ratio for total stroke was 0.48 (95% confidence interval 0.33–0.71) among men with a score ≥5 when compared with men with a score <3 (p for trend <0.001), adjusted for alcohol intake, physical activity, smoking and education. The corresponding hazard ratio in women was 1.07 (95% confidence interval 0.79–1.44). Adjustment for BMI, waist circumference and history of hypertension, hypercholesterolemia or diabetes did not appear to mediate the association (Model 2).

**Table 3:** Hazard ratios (HR) and 95% confidence intervals (CI) of total stroke by the Danish Dietary Guidelines Index.

	Men (n = 26 213)						
The Danish Dietary Guidelines Index	Cases, n	Model 1a*		Model 1b**		Model 2‡	
	1357	HR	95% CI	HR	95% CI	HR	95% CI
Score <3	386	1.00	Reference	1.00	Reference	1.00	Reference
Score 3-<4	659	0.73	0.65–0.83	0.79	0.70–0.90	0.79	0.70–0.90
Score 4-<5	284	0.57	0.49–0.66	0.67	0.57–0.78	0.66	0.56–0.77
Score ≥5	28	0.38	0.26–0.56	0.48	0.33–0.71	0.46	0.31–0.68
P for trend§		<0.001		<0.001		<0.001	
	Women (n = 28 837)						
The Danish Dietary Guidelines Index	Cases, n	Model 1a*		Model 1b**		Model 2‡	
	900	HR	95% CI	HR	95% CI	HR	95% CI
Score <3	99	1.00	Reference	1.00	Reference	1.00	Reference
Score 3-<4	408	0.98	0.79–1.23	1.12	0.89–1.39	1.11	0.89–1.38
Score 4-<5	310	0.69	0.55–0.87	0.87	0.69–1.10	0.86	0.68–1.08
Score ≥5	83	0.77	0.58–1.03	1.07	0.79–1.44	1.03	0.76–1.40
P for trend§		<0.001		0.158		0.104	

*Model 1a: adjusted for age and enrolment date.

**Model 1b: model 1a + adjusted for alcohol intake, physical activity, smoking and education.

^‡^Model 2: model 1b + adjusted for BMI, waist circumference, history of hypertension, hypercholesterolemia and diabetes.

^§^ p for trend was estimated by including the categorised index variable as a linear variable in the analysis.

### Associations with subtypes of stroke

**Fig 2:**
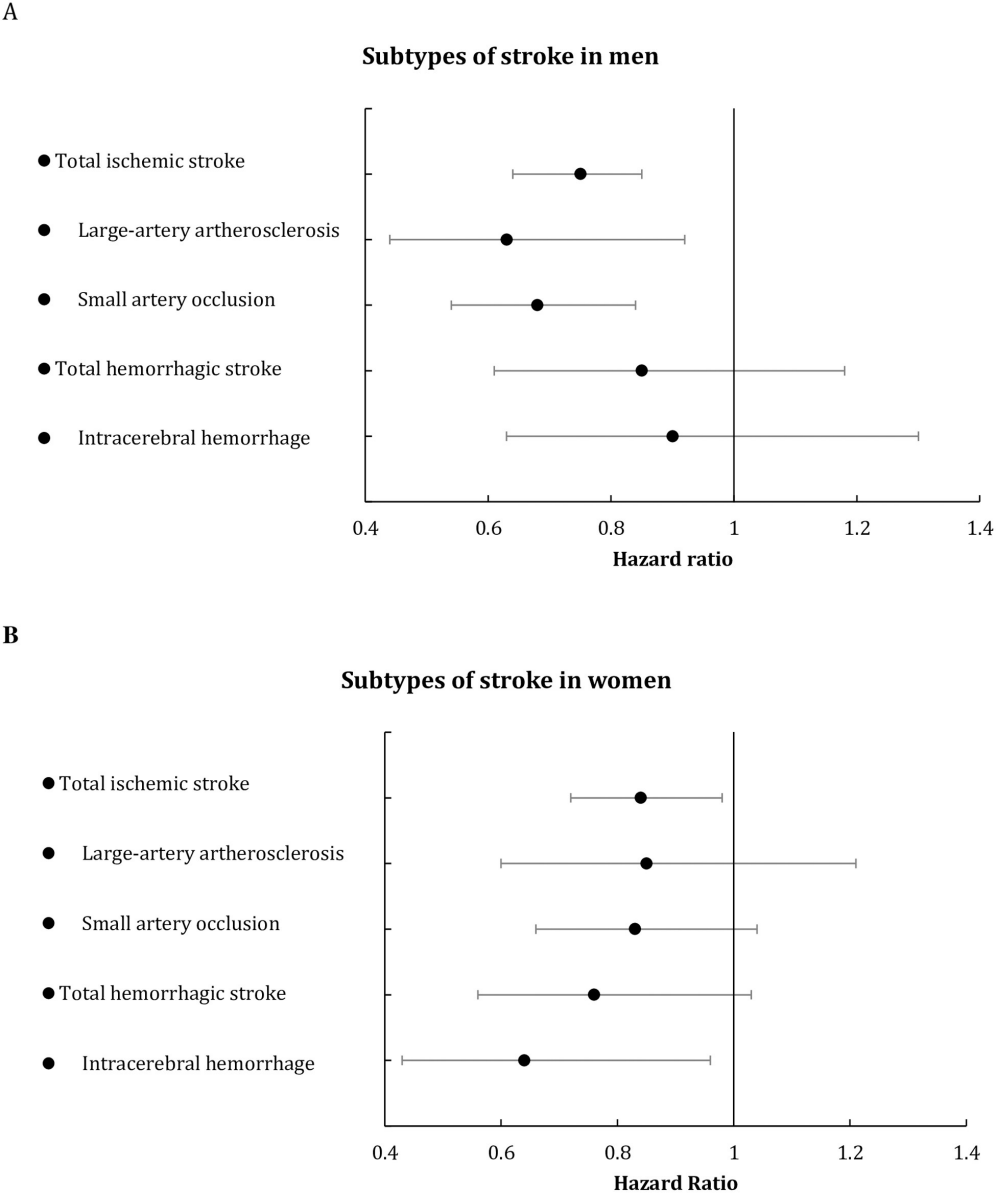
Hazard ratios and 95% confidence intervals from Cox proportional hazard models for subtypes of stroke. (A) Men with Danish Dietary Guidelines Index scores ≥4 compared to men with scores <4 and (B) women with Danish Dietary Guidelines Index scores ≥4 compared to women with scores <4, adjusted for alcohol intake, physical activity, smoking and education.

Fig 2 shows the adjusted results for subtypes of stroke in men and women. The hazard ratio for total ischemic stroke among men scoring ≥4 was 0.75 (95% confidence interval 0.65–0.86) compared with men scoring <4. Similar associations were seen for large-artery atherosclerosis (hazard ratio 0.63, 95% confidence interval 0.44–0.92) and small artery occlusion (hazard ratio 0.68, 95% confidence interval 0.54–0.84). For total hemorrhagic stroke and intracerebral hemorrhage tendencies of inverse associations were found.

In women, the hazard ratio of total ischemic stroke among those scoring ≥4 was 0.84 (95% confidence interval 0.72–0.98) compared with those scoring <4. Indications of inverse associations were found between the Index and large-artery atherosclerosis, small artery occlusion and total hemorrhagic stroke. The hazard ratio for intracerebral hemorrhage among women scoring ≥4 was 0.64 (95% confidence interval 0.43–0.96) compared with women scoring <4 (*P* = 0.030).

Results were similar for total stroke and total ischemic stroke when stratifying model 1b by educational level, BMI, smoking, history of hypertension and history of hypercholesterolemia (S3 and S4 Tables).

## Discussion

In this study, we found that higher adherence to the Danish food-based dietary guidelines was associated with lower rates of ischemic stroke among both men and women. In men, adherence to the Index was also inversely associated with total stroke, large-artery atherosclerosis and small artery occlusion, and tendencies towards inverse associations for total hemorrhagic stroke and intracerebral haemorrhage were observed. In women, adherence to the Index was inversely associated with intracerebral haemorrhage, while tendencies towards lower rates of large-artery atherosclerosis, small artery occlusion and total hemorrhagic stroke were found. No trend was observed for total stroke among women.

The strengths of our study include the follow-up design, the large number of validated cases, and almost complete follow-up for vital status. Only 298 individuals were censored due to emigration or changes in Civil Registration System numbers. Validation of stroke diagnoses was independent of dietary assessment, thus any misclassification of the outcome would only bias our results towards the null.

A potential limitation of the study is measurement error in self-reported dietary intake using questionnaires, but due to the cohort design, this misclassification is likely to be non-differential, leading to underestimation of the true association. Self-reported dietary assessment may also be affected by systematic error, [18] and it is possible that individuals with a low intake of healthy foods would tend to over-report their intake, thus achieving a higher score on the Danish Dietary Guidelines Index than their true score. However, misclassification of this type would also lead to underestimation of the true association.

Information about diet was only available at baseline and some participants may have changed their dietary habits during follow-up. If change is independent of risk of stroke, it will only lead to non-differential misclassification of the exposure over time. It is quite possible that adherence to the dietary guidelines improved over time mainly among those who perceive themselves to be at risk of stroke. In both cases, changes in dietary habits would lead to an attenuation of our results.

We adjusted extensively for potential confounders, and as adjustment altered estimates only slightly, concerns for residual confounding from these factors are minimal. However, residual confounding from other factors cannot be ruled out.

Although adjustment for total energy intake is common in studies of diet and disease, we did not adjust for total energy here, as the 2013 Danish food-based dietary guidelines are not expressed in terms of energy intake in the official pamphlet or on the associated website. Adjustment for total energy would therefore not reflect their adoption in the population. [7]

Few studies have investigated adherence to national dietary guidelines and incidence of stroke. A recent systematic review investigating diet quality as assessed by several diet indices, including the Healthy Eating Index (HEI), which measures adherence to the US dietary guidelines, found no reports of associations between HEI and incidence of stroke. [8] A study on the Dutch Healthy Diet Index, based on the Dutch national dietary guidelines, found no association with total stroke when comparing the highest and lowest quintiles of Index scores (hazard ratio 0.83, 95% confidence interval 0.66–1.04). [9] However, total stroke includes ischemic and hemorrhagic subtypes, which have different underlying etiologies [3,4] and thus may be differentially associated with diet. In our study, we observed stronger associations with ischemic stroke in men; the pattern was not so clear among women. In addition, there are some differences between the Dutch and other national guidelines. For example, the Dutch national guidelines recommend drinking filter coffee instead of coffee brewed by other methods. This is included as a component in the Dutch Healthy Diet Index, which further complicates comparison with our study. For this reason, it is important to consider the specific components included in national dietary guidelines when considering the generalizability of studies investigating their utility, as well as the characteristics of the study population investigated.

The Danish food-based dietary guidelines were developed using evidence from studies of single foods and nutrients, under the assumption that combining this evidence as a set of guidelines would be beneficial in primary prevention of disease, were the public to follow the guidelines. Our study tests this assumption, and indicates that the ensuing dietary pattern is indeed associated with lower rates of stroke, particularly ischemic stroke.

Our findings suggest that adherence to the Danish dietary guidelines is associated with lower rates of stroke, and the guidelines may thus be useful in primary prevention of disease.

## Supporting information

S1 Table Distribution of baseline characteristics according to the four categories of the Danish Dietary Guidelines Index (men). (DOCX)

S2 Table Distribution of covariates according to the four categories of the Danish Dietary Guidelines Index (women). (DOCX)

S3 Table Hazard ratios (HR) and 95% confidence intervals (CI) of total stroke by the Danish Dietary Guidelines Index stratified by educational level, BMI, smoking, history of hypertension and history of hypercholesterolemia in men and women. (DOCX)

S4 Table Hazard ratios (HR) and 95% confidence intervals (CI) of ischemic stroke by the Danish Dietary Guidelines Index stratified by educational level, BMI, smoking, history of hypertension and history of hypercholesterolemia in men and women. (DOCX)
