# Supplementary material for: Adherence to national food-based dietary guidelines and incidence of stroke: A cohort study of Danish men and women
Source: PLoS One. 2018 Oct 24;13(10):e0206242. doi: 10.1371/journal.pone.0206242 (PMC6200254; doi:10.1371/journal.pone.0206242)
Supplement: S1 Table — (DOCX) [file pone.0206242.s001.docx]

| **S1 Table.** Distribution of baseline characteristics according to the four categories of the Danish Dietary Guidelines Index (men). | | | | | | | | | | |
| --- | --- | --- | --- | --- | --- | --- | --- | --- | --- | --- |
|  |  |  | Score <3  (n=5693) | | Score 3-<4  (n=12 721) | | Score 4-<5  (n=6859) | | Score ≥5  (n=940) | |
|  | | | Median | 5th and 95th percentiles | Median | 5th and 95th percentiles | Median | 5th and 95th percentiles | Median | 5th and 95th percentiles |
| Age | | | 55.5 | 50.7-64.0 | 55.8 | 50.7-64.1 | 56.4 | 50.8-64.2 | 57.4 | 50.8-64.6 |
| Alcohol intake, g/day | | | 18.9 | 0.8-87.7 | 20.2 | 1.2-80.4 | 19.0 | 2.0-68.6 | 17.6 | 1.6-60.9 |
| BMI, (kg/m³) | | | 26.5 | 21.4-33.7 | 26.3 | 21.5-33.0 | 25.8 | 21.5-32.5 | 25.3 | 21.3-31.3 |
| Waist circumference, cm | | | 96.5 | 82.0-116.0 | 95.0 | 82.0-114.0 | 94.0 | 81.0-112.0 | 92.0 | 80.0-108.5 |
|  | | | n | % | n | % | n | % | n | % |
| Physical activity | | |  |  |  |  |  |  |  |  |
|  | <30 minutes/day | | 4053 | 71.2 | 8080 | 63.5 | 3719 | 54.2 | 412 | 43.8 |
|  | ≥30 minutes/day | | 1640 | 28.8 | 4641 | 36.5 | 3140 | 45.8 | 528 | 56.2 |
| Smoking | | |  |  |  |  |  |  |  |  |
|  | Never | | 1177 | 20.7 | 3269 | 25.7 | 2015 | 29.4 | 315 | 33.5 |
|  | Former | | 1481 | 26.0 | 4231 | 33.3 | 2921 | 42.6 | 434 | 46.2 |
|  | Current | | 3035 | 53.3 | 5221 | 39.0 | 1923 | 28.0 | 191 | 20.3 |
|  |  | <15 cigarettes/day | 569 | 10.0 | 1392 | 10.9 | 720 | 10.5 | 99 | 10.5 |
|  |  | 15-25 cigarettes/day | 1409 | 24.8 | 2330 | 18.3 | 778 | 11.3 | 64 | 6.8 |
|  |  | >25 cigarettes/day | 1057 | 18.6 | 1499 | 11.8 | 425 | 6.2 | 28 | 3.0 |
| Vocational or academic education | | | |  |  |  |  |  |  |  |
|  | None | | 838 | 14.7 | 1242 | 9.8 | 467 | 6.8 | 53 | 5.6 |
|  | <3 years | | 916 | 16.1 | 1748 | 13.7 | 789 | 11.5 | 106 | 11.3 |
|  | 3-4 years | | 2463 | 43.3 | 5430 | 42.7 | 2834 | 41.3 | 362 | 38.5 |
|  | >4 years | | 1476 | 25.9 | 4301 | 33.8 | 2769 | 40.4 | 419 | 44.6 |
| History of hypercholesterolemia | | | |  |  |  |  |  |  |  |
|  | Yes | | 371 | 6.5 | 966 | 7.6 | 739 | 10.8 | 165 | 17.6 |
|  | No | | 2724 | 47.9 | 5622 | 50.6 | 3560 | 51.9 | 470 | 50.0 |
|  | Don't know | | 2598 | 45.6 | 5315 | 41.8 | 2560 | 37.3 | 305 | 32.5 |
| History of hypertension | | |  |  |  |  |  |  |  |  |
|  | Yes | | 803 | 14.1 | 1809 | 14.2 | 1056 | 15.4 | 175 | 18.6 |
|  | No | | 3789 | 66.6 | 8841 | 69.5 | 4893 | 71.3 | 651 | 69.3 |
|  | Don't know | | 1101 | 19.3 | 2071 | 16.3 | 910 | 13.3 | 114 | 12.1 |
| History of diabetes | | |  |  |  |  |  |  |  |  |
|  | Yes | | 97 | 1.7 | 298 | 2.3 | 254 | 3.7 | 34 | 3.6 |
|  | No | | 5212 | 91.6 | 11 742 | 92.3 | 6326 | 92.2 | 871 | 92.7 |
|  | Don't know | | 379 | 6.7 | 681 | 5.4 | 681 | 5.4 | 35 | 3.7 |
| *Energy percentage | | |  |  |  |  |  |  |  |  |
