# Supplementary material for: Adherence to national food-based dietary guidelines and incidence of stroke: A cohort study of Danish men and women
Source: PLoS One. 2018 Oct 24;13(10):e0206242. doi: 10.1371/journal.pone.0206242 (PMC6200254; doi:10.1371/journal.pone.0206242)
Supplement: S2 Table — (DOCX) [file pone.0206242.s002.docx]

| **S2 Table.** Distribution of covariates according to the four categories of the Danish Dietary Guidelines Index (women). | | | | | | | | | | |
| --- | --- | --- | --- | --- | --- | --- | --- | --- | --- | --- |
|  |  |  | Score <3  (n=2680) | | Score 3-<4  (n=11 276) | | Score 4-<5  (n=12 062) | | Score ≥5  (n=2819) | |
|  | | | Median | 5th and  95th percentiles | Median | 5th and 95th percentiles | Median | 5th and 95th percentiles | Median | 5th and 95th percentiles |
| Age | | | 56.1 | 50.7-64.3 | 56.0 | 50.7-64.2 | 56.3 | 50.8-64.2 | 57.0 | 50.9-64.4 |
| Alcohol intake, g/day | | | 6.5 | 0.0-42.8 | 9.1 | 0.4-43.6 | 10.1 | 0.6-40.7 | 9.2 | 0.4-37.3 |
| BMI, (kg/m³) | | | 25.0 | 19.4-34.9 | 25.0 | 19.8-34.1 | 24.7 | 20.1-33.4 | 24.5 | 19.8-32.4 |
| Waist circumference, cm | | | 82.0 | 66.5-106.75 | 81.0 | 67.0-104.0 | 80.0 | 67.0-102.0 | 79.0 | 66.0-100.0 |
|  | | | n | % | n | % | n | % | n | % |
| Physical activity n (%) | | |  |  |  |  |  |  |  |  |
|  | <30 minutes/day | | 1933 | 72.1 | 7259 | 64.4 | 6617 | 54.9 | 1217 | 43.2 |
|  | ≥30 minutes/day | | 747 | 27.9 | 4017 | 35.6 | 5445 | 45.1 | 1602 | 56.8 |
| Smoking, n (%) | | |  |  |  |  |  |  |  |  |
|  | Never | | 864 | 32.2 | 4735 | 42.0 | 5641 | 46.8 | 1422 | 50.4 |
|  | Former | | 426 | 15.9 | 2318 | 20.6 | 3170 | 26.3 | 871 | 30.9 |
|  | Current | | 1390 | 51.8 | 4223 | 37.4 | 3251 | 26.9 | 526 | 18.7 |
|  |  | <15 cigarettes/day | 488 | 18.2 | 1785 | 15.8 | 1803 | 15.0 | 317 | 11.3 |
|  |  | 15-25 cigarettes/day | 730 | 27.2 | 2083 | 18.5 | 1266 | 10.5 | 184 | 6.5 |
|  |  | >25 cigarettes/day | 172 | 6.4 | 355 | 3.15 | 182 | 1.5 | 25 | 0.9 |
| Vocational or academic education, n (%) | | | |  |  |  |  |  |  |  |
|  | None | | 904 | 33.7 | 2466 | 21.9 | 1787 | 14.8 | 377 | 13.4 |
|  | <3 years | | 806 | 30.1 | 3609 | 32.0 | 3784 | 31.4 | 877 | 31.1 |
|  | 3-4 years | | 812 | 30.3 | 4147 | 36.8 | 4875 | 40.4 | 1138 | 40.4 |
|  | >4 years | | 158 | 5.9 | 1054 | 9.4 | 1616 | 13.4 | 427 | 15.2 |
| History of hypercholesterolemia, n (%) | | | |  |  |  |  |  |  |  |
|  | Yes | | 128 | 4.8 | 597 | 5.3 | 791 | 6.6 | 288 | 10.2 |
|  | No | | 1349 | 50.3 | 5622 | 49.9 | 6089 | 50.5 | 1477 | 52.4 |
|  | Don't know | | 1203 | 44.9 | 5057 | 44.9 | 5182 | 43.0 | 1054 | 37.4 |
| History of hypertension, n (%) | | |  |  |  |  |  |  |  |  |
|  | Yes | | 422 | 15.8 | 1934 | 17.2 | 2079 | 17.2 | 503 | 17.8 |
|  | No | | 1918 | 71.6 | 8104 | 71.9 | 8807 | 73.0 | 2089 | 74.1 |
|  | Don't know | | 340 | 12.7 | 1238 | 11.0 | 1176 | 9.8 | 227 | 8.1 |
| History of diabetes, n (%) | | |  |  |  |  |  |  |  |  |
|  | Yes | | 23 | 0.9 | 124 | 1.1 | 211 | 1.8 | 68 | 2.4 |
|  | No | | 2519 | 94.0 | 10726 | 95.1 | 11 379 | 94.3 | 2659 | 94.3 |
|  | Don't know | | 138 | 5.2 | 426 | 3.8 | 472 | 3.9 | 92 | 3.3 |
| *Energy percentage | | |  |  |  |  |  |  |  |  |
