# Supplementary material for: Adherence to national food-based dietary guidelines and incidence of stroke: A cohort study of Danish men and women
Source: PLoS One. 2018 Oct 24;13(10):e0206242. doi: 10.1371/journal.pone.0206242 (PMC6200254; doi:10.1371/journal.pone.0206242)
Supplement: S3 Table — (DOCX) [file pone.0206242.s003.docx]

**S3 Table.** Hazard ratios (HR) and 95% confidence intervals (CI) of total stroke by the Danish Dietary Guidelines Index stratified by educational level, BMI, smoking, history of hypertension and history of hypercholesterolemia in men and women.

|  |  | Score <3 | |  | Score 3-<4 | |  | Score 4-<5 | |  | Score ≥5 | |  | P for trend* |
| --- | --- | --- | --- | --- | --- | --- | --- | --- | --- | --- | --- | --- | --- | --- |
| Men | Cases,  n | HR | 95% CI |  | HR | 95% CI |  | HR | 95% CI |  | HR | 95% CI |  |  |
| Educational level |  |  |  |  |  |  |  |  |  |  |  |  |  |  |
| None | 185 | 1 | Reference |  | 0.87 | 0.63-1.19 |  | 0.79 | 0.50-1.24 |  | 0.77 | 0.24-2.46 |  | 0.904 |
| <3 years | 191 | 1 | Reference |  | 0.72 | 0.52-1.00 |  | 0.70 | 0.46-1.06 |  | 0.43 | 0.13-1.37 |  | 0.123 |
| 3-4 years | 560 | 1 | Reference |  | 0.78 | 0.64-0.95 |  | 0.71 | 0.56-0.91 |  | 0.53 | 0.30-0.97 |  | 0.005 |
| >4 years | 421 | 1 | Reference |  | 0.81 | 0.63-1.03 |  | 0.58 | 0.44-0.78 |  | 0.38 | 0.20-0.74 |  | 0.000 |
| BMI |  |  |  |  |  |  |  |  |  |  |  |  |  |  |
| <25 | 408 | 1 | Reference |  | 0.72 | 0.57-0.91 |  | 0.57 | 0.42-0.76 |  | 0.60 | 0.33-1.07 |  | 0.000 |
| 25-<30 | 694 | 1 | Reference |  | 0.85 | 0.71-1.02 |  | 0.75 | 0.60-0.93 |  | 0.53 | 0.31-0.93 |  | 0.007 |
| <30 | 255 | 1 | Reference |  | 0.84 | 0.63-1.12 |  | 0.82 | 0.57-1.18 |  | 0.14 | 0.02-1.04 |  | 0.261 |
| Smoking |  |  |  |  |  |  |  |  |  |  |  |  |  |  |
| Never | 276 | 1 | Reference |  | 0.76 | 0.56-1.03 |  | 0.71 | 0.50-1.00 |  | 0.28 | 0.11-0.71 |  | 0.040 |
| Former | 404 | 1 | Reference |  | 0.89 | 0.69-1.16 |  | 0.68 | 0.51-0.91 |  | 0.50 | 0.27-0.92 |  | 0.026 |
| Current | 677 | 1 | Reference |  | 0.77 | 0.65-0.91 |  | 0.64 | 0.51-0.81 |  | 0.69 | 0.37-1.26 |  | 0.000 |
| History of Hypertension |  |  |  |  |  |  |  |  |  |  |  |  |  |  |
| Yes | 334 | 1 | Reference |  | 0.83 | 0.63-1.08 |  | 0.69 | 0.50-0.95 |  | 0.49 | 0.24-0.97 |  | 0.025 |
| No | 798 | 1 | Reference |  | 0.80 | 0.68-0.94 |  | 0.60 | 0.48-0.74 |  | 0.46 | 0.28-0.77 |  | 0.000 |
| Don´t know | 225 | 1 | Reference |  | 0.73 | 0.54-1.00 |  | 0.92 | 0.63-1.33 |  | 0.43 | 0.13-1.38 |  | 0.484 |
| History of hypercholesterolemia | |  |  |  |  |  |  |  |  |  |  |  |  |  |
| Yes | 160 | 1 | Reference |  | 0.60 | 0.41-0.89 |  | 0.42 | 0.27-0.67 |  | 0.43 | 0.20-0.89 |  | 0.001 |
| No | 652 | 1 | Reference |  | 0.82 | 0.68-0.98 |  | 0.65 | 0.51-0.82 |  | 0.44 | 0.25-0.80 |  | 0.000 |
| Don´t know | 545 | 1 | Reference |  | 0.81 | 0.66-0.99 |  | 0.75 | 0.58-0.96 |  | 0.40 | 0.19-0.86 |  | 0.010 |
|  |  | Score <3 | |  | Score 3-<4 | |  | Score 4-<5 | |  | Score ≥5 | |  | P for trend* |
| Women | Cases | HR | 95% CI |  | HR | 95% CI |  | HR | 95% CI |  | HR | 95% CI |  |  |
| Educational level |  |  |  |  |  |  |  |  |  |  |  |  |  |  |
| None | 214 | 1 | Reference |  | 1.09 | 0.76-1.57 |  | 0.82 | 0.54-1.25 |  | 1.31 | 0.71-2.41 |  | 0.319 |
| <3 years | 289 | 1 | Reference |  | 1.01 | 0.68-1.49 |  | 0.76 | 0.50-1.15 |  | 1.06 | 0.64-1.79 |  | 0.152 |
| 3-4 years | 306 | 1 | Reference |  | 1.31 | 0.84-2.04 |  | 1.15 | 0.73-1.81 |  | 1.16 | 0.66-2.03 |  | 0.887 |
| >4 years | 91 | 1 | Reference |  | 1.24 | 0.49-3.17 |  | 0.74 | 0.28-1.92 |  | 0.77 | 0.26-2.32 |  | 0.077 |
| BMI |  |  |  |  |  |  |  |  |  |  |  |  |  |  |
| <25 | 448 | 1 | Reference |  | 1.30 | 0.94-1.81 |  | 1.09 | 0.77-1.53 |  | 1.27 | 0.82-1.97 |  | 0.840 |
| 25-<30 | 285 | 1 | Reference |  | 0.96 | 0.64-1.42 |  | 0.74 | 0.49-1.13 |  | 1.08 | 0.65-1.79 |  | 0.239 |
| <30 | 167 | 1 | Reference |  | 1.03 | 0.66-1.63 |  | 0.72 | 0.45-1.19 |  | 0.72 | 0.33-1.57 |  | 0.004 |
| Smoking |  |  |  |  |  |  |  |  |  |  |  |  |  |  |
| Never | 263 | 1 | Reference |  | 1.36 | 0.78-2.48 |  | 1.09 | 0.62-1.92 |  | 1.45 | 0.78-2.70 |  | 0.959 |
| Former | 186 | 1 | Reference |  | 0.92 | 0.52-1.60 |  | 0.69 | 0.39-1.21 |  | 0.78 | 0.40-1.49 |  | 0.090 |
| Current | 451 | 1 | Reference |  | 1.13 | 0.86-1.48 |  | 0.87 | 0.64-1.17 |  | 0.97 | 0.60-1.57 |  | 0.059 |
| History of Hypertension |  |  |  |  |  |  |  |  |  |  |  |  |  |  |
| Yes | 292 | 1 | Reference |  | 1.58 | 1.01-2.47 |  | 1.17 | 0.73-1.87 |  | 1.59 | 0.91-2.79 |  | 0.444 |
| No | 523 | 1 | Reference |  | 0.93 | 0.70-1.22 |  | 0.79 | 0.59-1.06 |  | 0.88 | 0.60-1.31 |  | 0.157 |
| Don´t know | 85 | 1 | Reference |  | 1.35 | 0.66-2.78 |  | 0.67 | 0.30-1.48 |  | 1.11 | 0.40-3.06 |  | 0.064 |
| History of hypercholesterolemia | |  |  |  |  |  |  |  |  |  |  |  |  |  |
| Yes | 87 | 1 | Reference |  | 1.66 | 0.70-3.96 |  | 0.89 | 0.36-2.19 |  | 1.51 | 0.57-3.99 |  | 0.489 |
| No | 444 | 1 | Reference |  | 1.12 | 0.82-1.53 |  | 0.87 | 0.62-1.21 |  | 1.07 | 0.70-1.64 |  | 0.217 |
| Don´t know | 369 | 1 | Reference |  | 1.04 | 0.74-1.47 |  | 0.89 | 0.62-1.27 |  | 0.90 | 0.55-1.49 |  | 0.110 |

Estimates are adjusted for age, enrolment date, alcohol intake, physical activity and smoking (Model 1b).

*p for trend was estimated by including the categorised index variable as a linear variable in the analysis.
