# Supplementary material for: Adherence to national food-based dietary guidelines and incidence of stroke: A cohort study of Danish men and women
Source: PLoS One. 2018 Oct 24;13(10):e0206242. doi: 10.1371/journal.pone.0206242 (PMC6200254; doi:10.1371/journal.pone.0206242)
Supplement: S4 Table — (DOCX) [file pone.0206242.s004.docx]

**S4 Table.** Hazard ratios (HR) and 95% confidence intervals (CI) of ischemic stroke by the Danish Dietary Guidelines Index stratified by educational level, BMI, smoking, history of hypertension and history of hypercholesterolemia in men and women.

|  |  | Score <4 | |  |  | Score ≥4 | |  |
| --- | --- | --- | --- | --- | --- | --- | --- | --- |
| Men | Cases,  n | HR | 95% CI |  |  | HR | 95% CI |  |
| Educational level |  |  |  |  |  |  |  |  |
| None | 158 | 1 | Reference |  |  | 0.71 | 0.45-1.13 |  |
| <3 years | 156 | 1 | Reference |  |  | 0.92 | 0.62-1.36 |  |
| 3-4 years | 487 | 1 | Reference |  |  | 0.77 | 0.62-0.96 |  |
| >4 years | 346 | 1 | Reference |  |  | 0.70 | 0.53-0.86 |  |
| BMI |  |  |  |  |  |  |  |  |
| <25 | 346 | 1 | Reference |  |  | 0.73 | 0.56-0.94 |  |
| 25-<30 | 586 | 1 | Reference |  |  | 0.77 | 0.63-0.94 |  |
| <30 | 215 | 1 | Reference |  |  | 0.88 | 0.63-1.23 |  |
| Smoking |  |  |  |  |  |  |  |  |
| Never | 235 | 1 | Reference |  |  | 0.81 | 0.61-1.08 |  |
| Former | 330 | 1 | Reference |  |  | 0.73 | 0.58-0.93 |  |
| Current | 582 | 1 | Reference |  |  | 0.71 | 0.56-0.89 |  |
| History of Hypertension |  |  |  |  |  |  |  |  |
| Yes | 273 | 1 | Reference |  |  | 0.71 | 0.54-0.95 |  |
| No | 686 | 1 | Reference |  |  | 0.68 | 0.57-0.82 |  |
| Don´t know | 188 | 1 | Reference |  |  | 1.05 | 0.75-1.49 |  |
| History of hypercholesterolemia | |  |  |  |  |  |  |  |
| Yes | 128 | 1 | Reference |  |  | 0.55 | 0.37-0.82 |  |
| No | 558 | 1 | Reference |  |  | 0.77 | 0.63-0.94 |  |
| Don´t know | 461 | 1 | Reference |  |  | 0.76 | 0.60-0.96 |  |
|  |  | Score <4 | |  |  | Score ≥4 | |  |
| Women | Cases | HR | 95% CI |  |  | HR | 95% CI |  |
| Educational level |  |  |  |  |  |  |  |  |
| None | 173 | 1 | Reference |  |  | 0.95 | 0.69-1.31 |  |
| <3 years | 234 | 1 | Reference |  |  | 0.78 | 0.60-1.02 |  |
| 3-4 years | 233 | 1 | Reference |  |  | 0.90 | 0.69-1.18 |  |
| >4 years | 73 | 1 | Reference |  |  | 0.69 | 0.43-1.10 |  |
| BMI |  |  |  |  |  |  |  |  |
| <25 | 338 | 1 | Reference |  |  | 0.96 | 0.77-1.20 |  |
| 25-<30 | 233 | 1 | Reference |  |  | 0.80 | 0.62-1.05 |  |
| <30 | 142 | 1 | Reference |  |  | 0.72 | 0.51-1.03 |  |
| Smoking |  |  |  |  |  |  |  |  |
| Never | 214 | 1 | Reference |  |  | 0.86 | 0.66-1.13 |  |
| Former | 143 | 1 | Reference |  |  | 0.76 | 0.55-1.07 |  |
| Current | 356 | 1 | Reference |  |  | 0.85 | 0.68-1.07 |  |
| History of Hypertension |  |  |  |  |  |  |  |  |
| Yes | 246 | 1 | Reference |  |  | 0.89 | 0.69-1.16 |  |
| No | 406 | 1 | Reference |  |  | 0.87 | 0.71-1.07 |  |
| Don´t know | 61 | 1 | Reference |  |  | 0.50 | 0.29-0.87 |  |
| History of hypercholesterolemia | |  |  |  |  |  |  |  |
| Yes | 67 | 1 | Reference |  |  | 0.64 | 0.39-1.05 |  |
| No | 355 | 1 | Reference |  |  | 0.80 | 0.64-1.00 |  |
| Don´t know | 291 | 1 | Reference |  |  | 0.95 | 0.75-1.21 |  |

Estimates are adjusted for age, enrolment date, alcohol intake, physical activity and smoking (Model 1b).
